# Neural cues differentially modulate colorectal cancer cell behavior depending on patients’ genomic background

**DOI:** 10.1016/j.isci.2026.116153

**Published:** 2026-05-28

**Authors:** Meike S. Thijssen, Rosaria Chilà, Giovanni Crisafulli, Kim M. Smits, Alberto Bardelli, Werend Boesmans, Veerle Melotte

**Affiliations:** 1 Department of Pathology, GROW – Research Institute for Oncology and Reproduction, Maastricht University Medical Center, Maastricht, the Netherlands; 2 Biomedical Research Institute (BIOMED), Hasselt University, Hasselt, Belgium; 3 IFOM ETS - The AIRC Institute of Molecular Oncology, Milan, Italy; 4 Department of Oncology, Molecular Biotechnology Center, University of Torino, Turin, Italy

**Keywords:** oncology, microenvironment, neuroscience

## Abstract

While neurons are mostly described as pro-tumorigenic and linked with a poor prognosis, differing outcomes have been reported for colorectal cancer (CRC) due to the lack of control for neural and patient subtype diversity. In this study, we investigated the effect of neural cues on patient-derived CRC cell lines selected based on genomic status, e.g., microsatellite instability (MSI) and *KRAS* and *BRAF* mutations. Although most neural signals increased clonogenicity, the adrenergic neurotransmitter epinephrine had the opposite effect. Epinephrine also decreased CRC cell viability, independent of the genomic status. Vasoactive intestinal peptide decreased cell viability only in *BRAF* wild-type cells. Interestingly, all neural signals induced migration in microsatellite stable (MSS) cells, with no effect in cells with MSI. Epinephrine or glial cell line-derived neurotrophic factor also stimulated migration specifically in *BRAF*-mutated cells. These results emphasize the importance of targeting specific neural signaling pathways and highlight that patient stratification is essential for cancer neuroscience studies.

## Highlights

• Neural cues differentially regulate clonogenicity and migration in CRC
• Adrenergic signaling suppresses proliferation while enhancing migration in CRC
• Neural-induced migration depends on genomic subtype and is most evident in MSS CRC
• Baseline transcriptomic states predict CRC responsiveness to neural signals

## Graphical abstract

**Figure:**
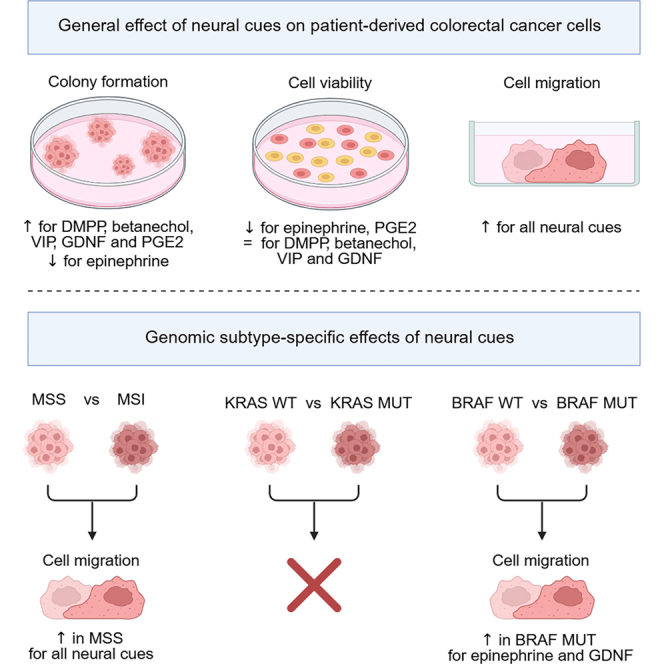

## Introduction

The nervous system has been established as a key regulator of carcinogenesis, adding a neural dimension to the tumor microenvironment (TME), which comprises endothelial and immune cells, fibroblasts, and extracellular matrix (ECM) components.^1^ The TME plays an important role in shaping tumor behavior, progression, and therapy responses, a concept well established for immune cells but less understood for neural components.^2^ In colorectal cancer (CRC), which is a heterogeneous disease that develops from epithelial cells undergoing a cascade of genetic and epigenetic alterations,^3^ such neural influences are only beginning to be explored.

The colonic epithelium is highly innervated, with different types of both gut extrinsic and intrinsic neurons as well as enteric glia projecting into the colonic mucosa.^4^ Several of these neuronal subtypes have already been described to play a role in cancer, with some of their commonly secreted neurotransmitters (e.g., acetylcholine and epinephrine) being important in the context of CRC.^5^ We previously showed that the colonic tumor tissue is mostly innervated by vasoactive intestinal peptide (VIP)-immunoreactive neuronal processes,^6^ suggesting that VIP is released close to tumor cells. Also, enteric glial cells, known to secrete molecules such as prostaglandin E2 (PGE2) and glial cell line-derived neurotrophic factor (GDNF),^7^^,^^8^ can be found in the tumor tissue and show signs of reactivity in CRC.^9^ While the overall idea is that neurons are pro-tumorigenic and linked to a poor prognosis, denervation studies and neurotransmitter signaling interventions indicate contradicting effects in CRC.^5^^,^^10^^,^^11^ This could be because these studies focused on different neural subtypes, and the models do not reflect the variety of CRC patient subtypes.

Although many distinct mutational events have been characterized in CRC, mutational classifications have proven useful in the stratification of CRC patients for prognosis and treatment responses. One of these classifications involves the microsatellite stability status of a tumor, which is measured by the accumulation of modifications in short repetitive sequences of DNA.^12^ In mismatch repair (MMR)-deficient tumors, frequently occurring modifications in microsatellites are not repairable, which leads to a high mutational burden, and these tumors are classified as microsatellite unstable (MSI). In CRC, only around 15% of tumors are categorized as being MSI, whereas the majority are microsatellite stable (MSS) tumors.^13^ Because MSI tumors are linked to a high mutational burden and, therefore, have a high neo-antigen load, these tumors are often referred to as immunologically hot, with the TME being infiltrated by effector immune cells.^14^^,^^15^^,^^16^ Therapy responses are strongly affected by the microsatellite stability status, as it has been shown that neoadjuvant chemotherapy does not induce a response in most MSI tumors, whereas these tumors are highly responsive to immune checkpoint blockade (ICB) therapies, in contrast to MSS tumors.^17^

Additional to this broad genomic subtyping, mutations in specific genes are also used to further stratify CRC patients. These are mutations observed only in a subset of CRC patients, such as mutations in the *KRAS* and *BRAF* oncogenes, which arise when aberrant foci progress into adenomas.^12^^,^^18^^,^^19^^,^^20^ Both genes are members of the RAS/RAF/MAPK pathway, which is essential in the regulation of cell proliferation, differentiation, and apoptosis.^21^^,^^22^ Mutations in *KRAS* and *BRAF* are followed by other mutations that initiate the adenoma to carcinoma sequence, such as those in *PIK3CA*, *TP53*, and loss of *18q* and *SMAD4*, which define a smaller group of patients.^12^^,^^18^^,^^19^ Around 40% of sporadic CRC patients are affected by *KRAS* mutations, which mostly occur in codons 12 and 13.^23^ *KRAS* mutations are not exclusively linked to the microsatellite status but are more common in MSS tumors.^21^ *BRAF* mutations occur in about 10% of sporadic CRC cases and are condensed to exon 15 with the allelic variant p.V600E.^24^ *BRAF* mutations are highly enriched in MSI tumors and, in most cases, are mutually exclusive with *KRAS* mutations.^25^ Importantly, *KRAS*- and *BRAF*-mutated tumors are largely unresponsive to epidermal growth factor receptor (EGFR)-targeted therapies (cetuximab and panitumumab), as they are independent downstream activators in the EGFR pathway.^26^^,^^27^ Thus, *KRAS* and *BRAF* mutation status is an important factor for determining clinical treatment strategies.

Despite this well-established genomic diversity, which is already used in clinical practice, it remains unknown whether neural cues differentially affect patients carrying tumors with a characteristic molecular background. In other words, it is unclear whether the neural component of the TME contributes differently to disease behavior across molecular CRC subtypes. In this study, we address this question by exposing a cohort of patient-derived CRC cell lines, selected based on their defined genomic profiles (MSI/MSS and *KRAS*/*BRAF* mutation status), to a panel of neural cues. This approach allowed us to explore whether patient-specific genetic backgrounds determine how CRC cells respond to neural signaling, thereby linking cancer neuroscience to clinically relevant molecular diversity in CRC.

## Results

### Neural signaling factors affect CRC cell proliferation and migration

**Figure 1:**
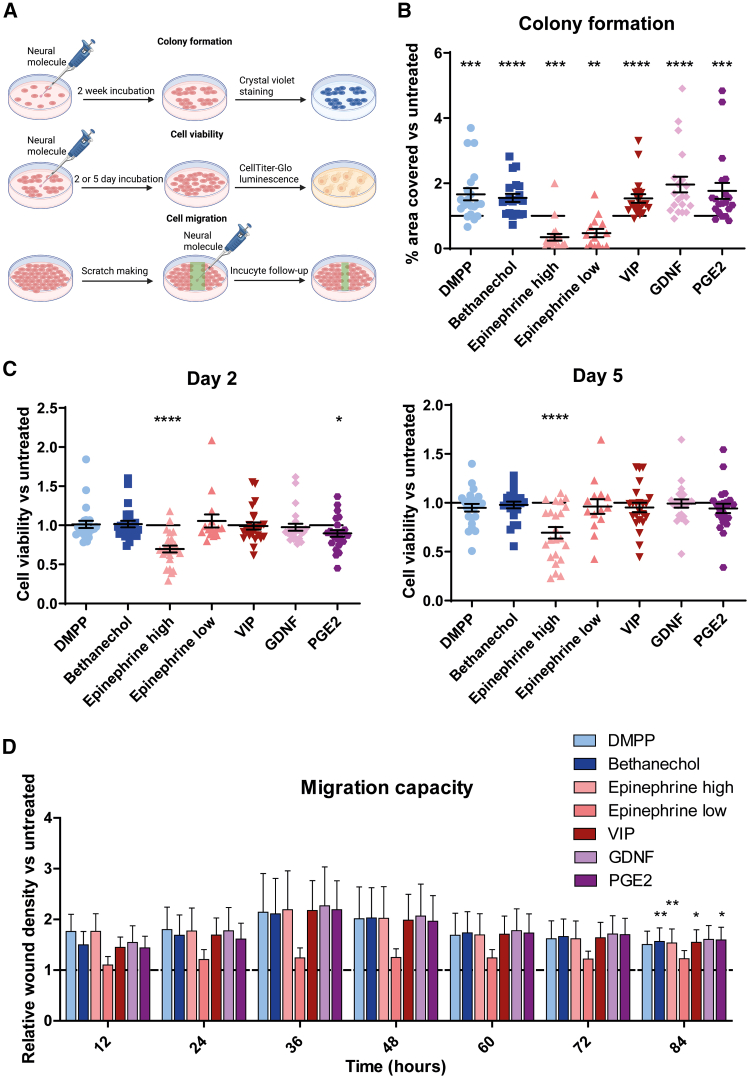
The effect of neural cues on CRC cell characteristics (A) Patient-derived CRC cell lines were treated with the neural cues DMPP, bethanechol, epinephrine, VIP, GDNF, and PGE2. The effects of neural stimulation were characterized by colony-formation assays, cell viability assays, and scratch migration assays. (B) DMPP, bethanechol, VIP, GDNF, and PGE2 increased the colony-formation ability of CRC cells, while epinephrine reduced the colony outgrowth. (C) Epinephrine (high concentration) significantly decreased the viability of CRC cells after both two and five days of treatment, and PGE2 slightly reduced cell viability, while the other neural cues did not affect CRC cell viability. (D) Migration capacity seems to increase after stimulation with neural molecules compared to untreated cells at most time points, but the effect was not significant. *N* = 20–24 cell lines, represented as dots; and *n* = 3 replicates per cell line. All data are presented as the mean ± SEM, and significance between untreated and treated cells was analyzed using Wilcoxon matched-pair signed-rank tests: ∗*p* < 0.05, ∗∗*p* < 0.01, ∗∗∗*p* < 0.001, and ∗∗∗∗*p* < 0.0001.

First, to evaluate the general impact of neural cues across all CRC subtypes, we tested the effect of various neural signals on the total panel of 22 patient-derived CRC cell lines representing diverse genetic backgrounds (Figure 1A). Adrenergic (mostly ADRA2A and ADRA2C), nicotinic cholinergic (mostly CHRNA5 and CHRNB1), VIPergic (mostly VIPR1), PGE2 (mostly PTGER4), and, to a lower extent, muscarinic cholinergic (mostly CHRM1) receptors were found to be expressed by most CRC cell lines tested, while GDNF receptors were not commonly expressed (Figure S1). The concentrations of dimethylphenylpiperazinium (DMPP; a nicotinic receptor agonist), bethanechol (a muscarinic receptor agonist), epinephrine, VIP, GDNF, and PGE2 were chosen based on literature and pilot cell viability experiments with two different CRC cell lines (MSS vs. MSI). For the latter, concentrations were chosen based on a possible effect of the treatment and/or a different response between the two cell lines at day 5 after treatment (Figure S2). For epinephrine, an additional concentration (20 μM) was selected, as the higher concentration (200 μM) had a viability-reducing effect on both cell lines, which could mask possible differences between the CRC groups. Colony formation was found to be increased after DMPP, bethanechol, VIP, GDNF, and PGE2 treatments compared with untreated CRC cells (Figure 1B), indicating that signaling factors typically released by neurons and glial cells can promote clonogenicity. In contrast, epinephrine decreased the colony-formation ability of the same CRC cell lines. Furthermore, a high epinephrine concentration reduced cell viability compared with untreated cells both after two and five days of treatment. PGE2 also slightly decreased cell viability, while the other neural cues did not affect overall cell viability (Figure 1C). Although there was a high variability between the cell lines, the migratory capacity of CRC cells tended to increase after all neural treatments, with the effects being significant for bethanechol, epinephrine (high concentration), VIP, and PGE2 after 84 h (Figure 1D). These results suggest that epinephrine has a substantial effect on CRC cell proliferation as it decreased both cell viability and colony-formation ability. Moreover, the effect appears to be concentration dependent because a lower concentration of epinephrine had a less pronounced effect. Both glial-derived molecules GDNF and PGE2 positively affected colony formation but had no effect and even a slightly reducing effect on CRC cell viability, respectively. In addition, the stimulating effect of DMPP, bethanechol, and VIP on colony formation confirmed a possible role of cholinergic and VIPergic signaling in CRC.^5^^,^^6^^,^^28^

To understand the effect of neural cues on different CRC patient subtypes, the cell lines were divided into 4 clinically relevant subgroups: control (MSS, *KRAS* wild-type [WT], and *BRAF* WT), MSI (MSI, *KRAS* WT, and *BRAF* WT), *KRAS* MUT (MSS, *KRAS* MUT, and *BRAF* WT), and *BRAF* MUT (MSS, *KRAS* WT, and *BRAF* MUT) (Table 1). The results showed that the reduced colony-formation capacity after epinephrine treatment was mainly observed in the control, MSI, and *BRAF*-mutated groups. Interestingly, bethanechol, VIP, and GDNF upregulated the clonogenicity specifically in the control group. Next to the epinephrine-induced decrease in cell viability, significant for the control and MSI groups, GDNF decreased cell viability specifically in MSI lines, and PGE2 reduced cell viability only in the control group. Assessing CRC cell migration, no significant differences between the treated and untreated conditions could be identified for all groups at 84 h, which was the most affected time point, but the variability between cell lines was high. Together, these results suggest that genomic profiles of CRC lines affect the response to neural signals.

**Table 1:** The effect of neural cues based on molecular CRC subtypes

	DMPP	Bethanechol	Epinephrine	VIP	GDNF	PGE2
**Colony formation**						

Control	–	↑ (∗∗)	↓ (∗∗)	↑ (∗)	↑ (∗)	–
MSI	–	–	↓ (∗∗)	–	–	–
*KRAS* MUT	–	–	–	–	–	–
*BRAF* MUT	–	–	↓ (∗∗)	–	–	–

**Cell viability**						

Control	–	–	↓ (∗∗)	–	–	↓ (∗∗)
MSI	–	–	↓ (∗∗)	–	↓ (∗)	–
*KRAS* MUT	–	–	–	–	–	–
*BRAF* MUT	–	–	–	–	–	–

**Cell migration**						

Control	–	–	–	–	–	–
MSI	–	–	–	–	–	–
*KRAS* MUT	–	–	–	–	–	–
*BRAF* MUT	–	–	–	–	–	–

The treated CRC cell lines were divided into 4 genomic groups: control (MSS, *KRAS* WT, and *BRAF* WT; *N* = 9–10 depending on the assay), MSI (MSI, *KRAS* WT, and *BRAF* WT; *N* = 3–5), *KRAS* MUT (MSS, *KRAS* MUT, and *BRAF* WT; *N* = 3–5), and *BRAF* MUT (MSS, *KRAS* WT, and *BRAF* MUT; *N* = 3–4). Effect of the different neural signals on colony formation, cell viability, and cell migration are displayed per genomic group. Arrows signify an increase (↑) or decrease (↓), and stars represent the significance level analyzed by two-way ANOVA with Dunnett’s multiple comparison correction (∗*p* < 0.05; ∗∗*p* < 0.01).

### Microsatellite status influences the effect of neural signaling on CRC cell migration

**Figure 2:**
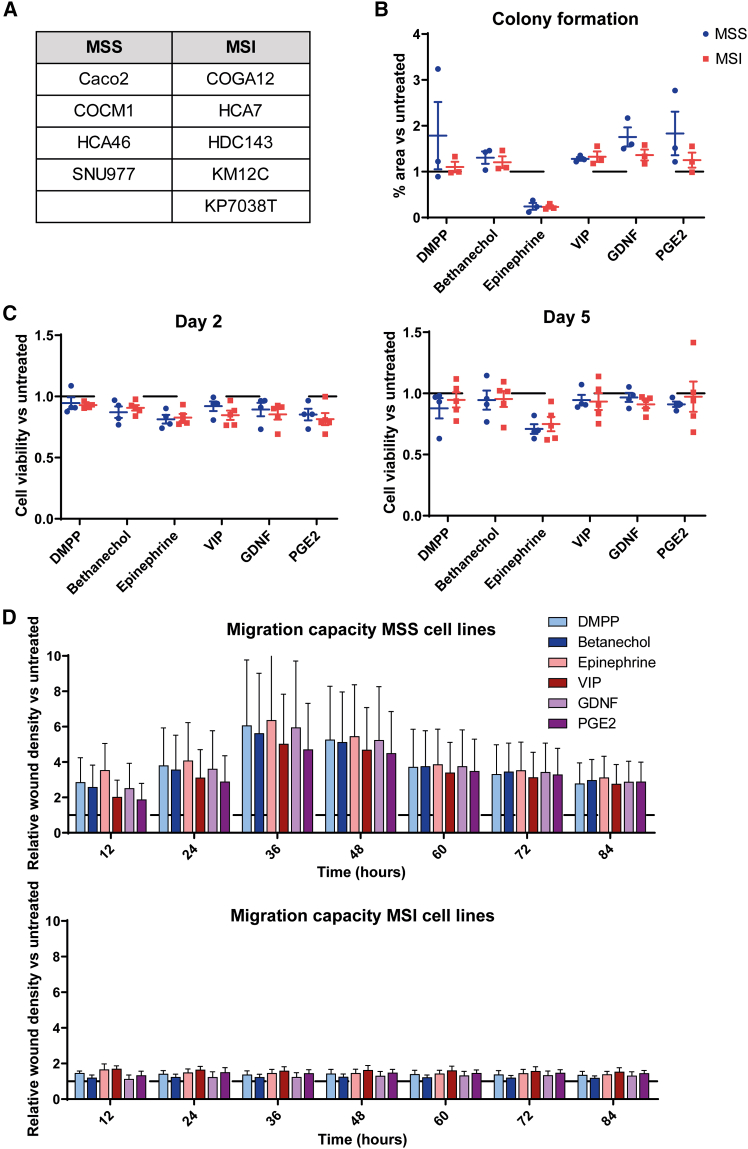
The role of microsatellite status in the response to neural cues (A) An MSS (*N* = 4) and an MSI (*N* = 5) cell line cohorts were selected. (B) The colony-formation ability was reduced in the epinephrine group but not affected by the MSI status. (C) Cell viability of CRC cells after neural stimulation was similarly affected in MSS and MSI cell lines. (D) Migration capacity seems to be increased in MSS cell lines compared with MSI cell lines for all neural signals. All data are presented as the mean ± SEM. Unpaired *t* tests were used to compare MSS (upper graph) and MSI (lower graph) cell lines for the different neural signals.

To investigate whether MSS and MSI CRC cells respond differently to neural cues, we selected two cohorts of five cell lines each (Figure 2A). The cohorts were matched for tumor location, mutational profile, neuroreceptor expression, and culture conditions to minimize confounding variables. MSS and MSI cell lines responded similarly to all neural signals for clonogenicity (Figure 2B). Furthermore, there was no difference in response between MSS and MSI cell lines in terms of cell viability (Figure 2C). Interestingly, the migration capacity seemed to be affected by MSI status: only MSS cell lines showed increased scratch closure in response to the different neural cues, whereas MSI cell lines did not display any notable migration changes (Figure 2D). These results were similar when adding the tested cell lines from other cohorts (*KRAS* WT and *BRAF* WT groups) to the MSS group (Figure S3). MSS cell lines presented with a significantly higher expression of ADRA2C and lower expression of VIPR1 compared with MSI cell lines (Figure S1A). However, other neural signals also tended to increase the migration of the MSS cell lines, without significant differences in their respective receptor expression. Transcriptomic analysis further indicated coordinated enrichment of the pathways linked to ECM interaction, lipid metabolism, and PPAR signaling in MSS cells (Table S1). These pathways support cytoskeletal remodeling, membrane dynamics, and metabolic flexibility, which are essential for active cell migration, suggesting that enhanced migration in MSS cells is driven by metabolic-ECM programs, rather than classical EMT gene expression. Overall, these findings indicate that MSS cells may be more responsive to neural signals affecting migratory behavior, indicating a possible interaction between the microsatellite status and neural input in shaping CRC metastatic potential.

### *KRAS* and *BRAF* mutation status affects CRC cell responses to specific neural cues

**Figure 3:**
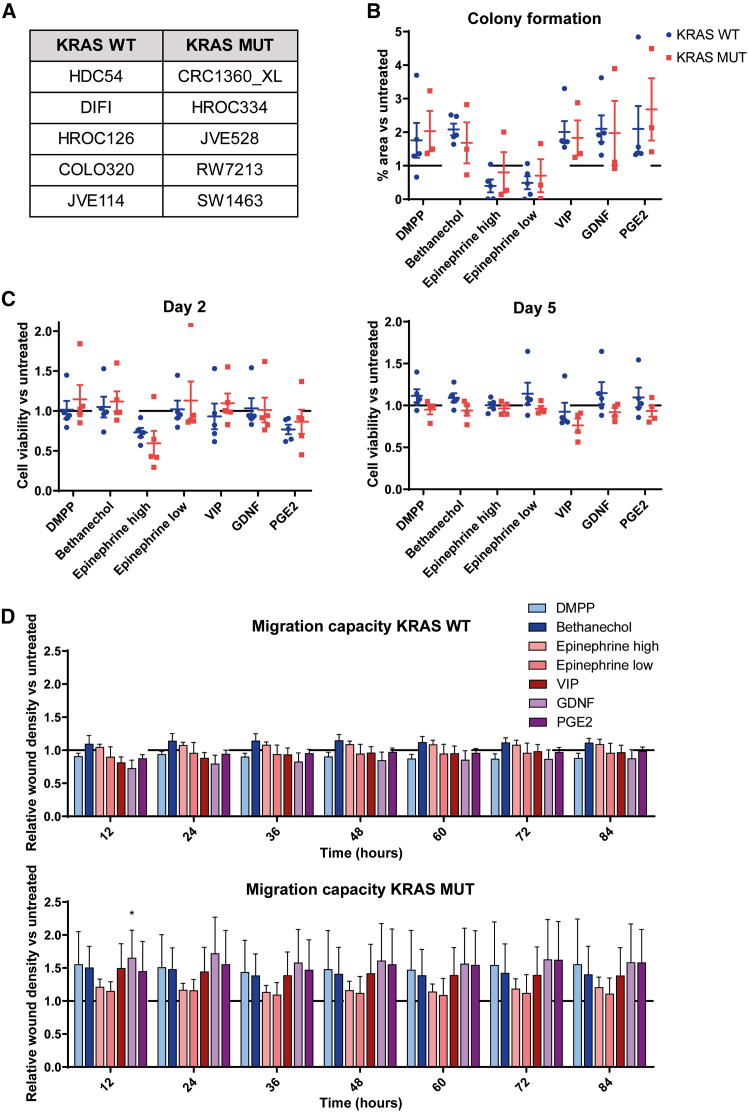
The role of *KRAS* mutation status in response to neural cues (A) A *KRAS* WT (*N* = 5) and a *KRAS*-mutated (*N* = 5) cell line cohorts were selected. (B) PGE2 increased the clonogenicity of CRC cells in this cohort, but *KRAS* mutation status did not alter the neural responses. (C) Cell viability was not linked to *KRAS* mutation status. (D) *KRAS*-mutant cell lines showed a trend of increased migration in the DMPP, GDNF, and PGE2 conditions compared with *KRAS* WT cell lines, although only slightly. All data are presented as the mean ± SEM. Statistical analysis was performed using unpaired *t* tests to compare *KRAS* WT (upper graph) and *KRAS* MUT (lower graph) cell lines for the different neural signals.

To assess whether *KRAS* mutation status impacts the effect of neural cues, two cohorts of patient-derived CRC cell lines—five *KRAS* WT and five *KRAS*-mutant lines—matched for tumor location, additional mutational features, and culture conditions—were selected (Figure 3A). *KRAS* mutation status was not associated with clonogenicity in response to neural cues in this cohort (Figure 3B). *KRAS* mutation status also did not alter the effects of neural cues on cell viability (Figure 3C). While a trend could be observed for DMPP, GDNF, and PGE2 toward an increased cell migration in *KRAS*-mutant cell lines, the difference was small and the variability between the cell lines was high (Figure 3D). These trends were not observed anymore when the cohort was expanded with the cell lines tested in other cohorts (MSS and *BRAF* WT groups) to the *KRAS* WT group (Figure S4). *KRAS* mutation status was linked to an increase in CHRNB1 expression and a decrease in VIPR1 expression, possibly explaining the effect of the nicotinic activator DMPP on CRC cell migration. However, expression data did not correlate with the small effect of other neural signals (Figure S1B). Also, transcriptomic analyses did not identify significant differentially expressed genes or affected pathways between the *KRAS* cohorts, consistent with the small trends in functional assays.

**Figure 4:**
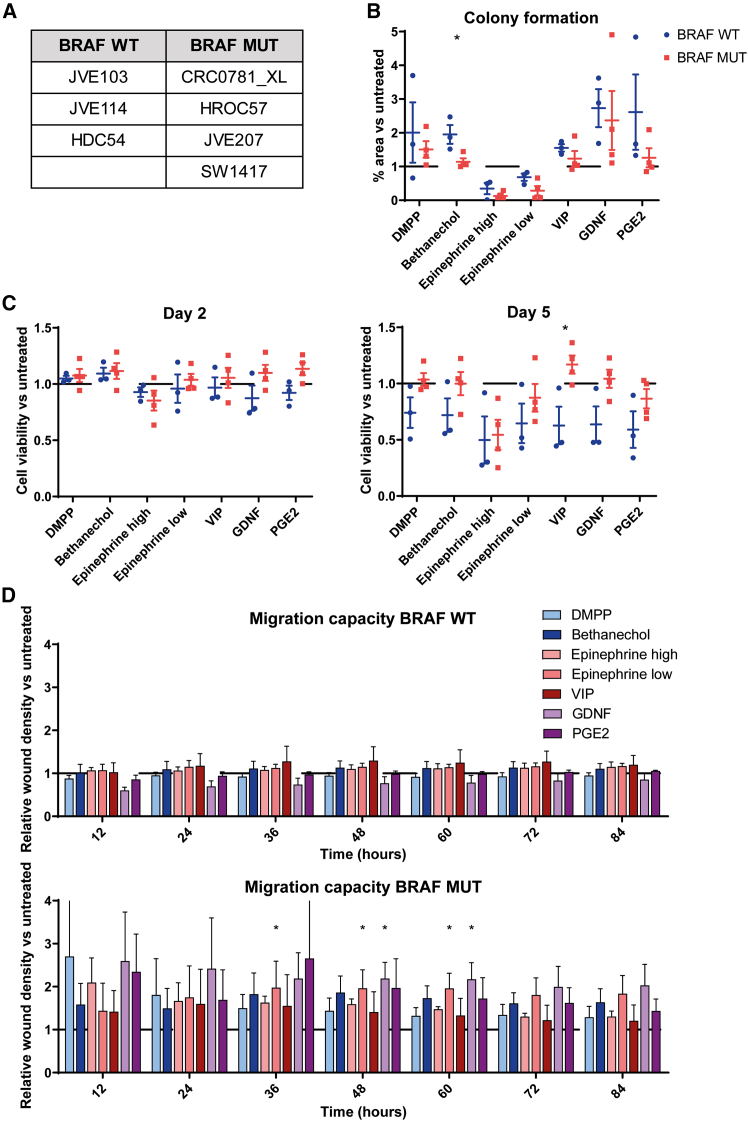
The role of *BRAF* mutation status in the response to neural cues (A) A *BRAF* WT (*N* = 3) and a *BRAF*-mutated (*N* = 4) cell line cohorts were selected. (B) Bethanechol increased the clonogenicity of *BRAF* WT CRC cells compared with *BRAF*-mutated cell lines. (C) VIP-treated *BRAF* WT cells presented with reduced cell viability compared with *BRAF*-mutated cell lines after five days. (D) *BRAF*-mutant cell lines showed a trend of increased migration in the epinephrine (low) and GDNF conditions compared with *BRAF* WT cell lines. All data are presented as the mean ± SEM. Statistical analysis was performed using unpaired *t* tests to compare *BRAF* WT (upper graph) and *BRAF* MUT (lower graph) cell lines for the different neural signals (∗*p* < 0.05; ∗∗*p* < 0.01).

Similarly, to investigate whether patients with *BRAF* mutation status respond differently to neural cues, a cohort of three *BRAF* WT and four *BRAF*-mutated patient cell lines was selected (Figure 4A). Clonogenicity seemed to be affected by *BRAF* mutation status upon muscarinic receptor agonism, as only *BRAF* WT cells were able to increase their colony-formation ability after bethanechol stimulation (Figure 4B). Limited by the high variability between cell lines, cell viability was also altered by *BRAF* mutation status for multiple neural cues after 5 days, although only the results after VIP treatment proved to be significant (Figure 4C). Moreover, the migration phenotype was affected by *BRAF* mutation status, where a long-term increase by epinephrine (low concentration) and GDNF treatments was observed in *BRAF*-mutated compared with *BRAF* WT cell lines (Figure 4D). Interestingly, the described colony formation, cell viability, and epinephrine-stimulated migration effects were observed even after the addition of extra cell lines tested in the other cohorts (MSS and *KRAS* WT groups) to the *BRAF* WT group (Figure S5). In this cohort, the increase in ADRA2C was observed, which might explain the increase in migration by epinephrine treatment (Figure S1C). Transcriptomic comparison between *BRAF*-mutated and *BRAF* WT CRC cell lines revealed trends toward altered metabolic and detoxification programs as well as the enrichment of pathways involved in cell surface and glycosaminoglycan biosynthesis (Table S2). This suggests that the *BRAF* mutation status does not impose a dominant intrinsic migratory program, but rather modulates the cellular state and responsiveness to external cues. Consistent with this, *BRAF*-mutated cells exhibited modest increases in migration and viability only under specific experimental conditions, indicating a context-dependent phenotype.

Taken together, our findings indicated that while inter-cell line variability was high, the mutational status of the patient from whom the cell line was derived significantly influenced CRC cell responses to neural cues.

## Discussion

While neurons are mostly described as pro-tumorigenic, multiple studies targeting neural signaling in CRC have reported contradictory findings.^5^^,^^29^ The fact that distinct neural interventions and different CRC subtypes were used in these studies is often not considered. However, CRC patient subtypes, as based on genomic status, are differentially associated with prognosis as well as treatment responses and are, therefore, crucial for making decisions on cancer patient management.^30^ Furthermore, the colon is highly innervated by various neural subtypes from both the parasympathetic and sympathetic as well as the intrinsic enteric nervous system, and, therefore, colonic tumors can receive a plethora of neural cues. In this study, we used a panel of neural stimuli to model the potential effect of different neural cells on CRC characteristics in terms of clonogenicity, cell viability, and migration capacity. Furthermore, we applied these neural cues to cohorts of patient-derived CRC lines that were selected based on genomic status, e.g., MSI, *KRAS* mutated, and *BRAF* mutated, to understand which subtypes of CRC patients respond to neural signals. We show that neural cues affect these CRC cell characteristics. Moreover, our data suggest that the genomic subtype of patient-derived CRC cells can influence their response to neural signals. Overall, epinephrine showed a pronounced effect on CRC cell proliferation. Interestingly, the other neural molecules increased clonogenicity, and epinephrine as well as the other neural signals were found to stimulate cell migration. This suggests that epinephrine might have both a tumor-suppressing role in terms of proliferation and a promoting role on metastasis, similar to cholinergic, VIPergic, and enteric glial signals. The adrenergic influence on cell viability and migration appears to be concentration dependent, as a lower concentration did diminish the effect.

In line with our data, another study also observed reduced proliferation upon epinephrine incubation,^31^ while few other studies have shown that epinephrine boosts CRC cell growth and metastasis.^32^^,^^33^^,^^34^^,^^35^ While these conflicting results could be caused by the use of different epinephrine concentrations, variations in the CRC cell lines used across studies may also have contributed. Most of these studies have used only HT-29 cells, but we observed that not all patient-derived CRC lines respond in the same way. HT-29 is *BRAF* mutated and MSS, and cell lines within this group (*BRAF* MUT in Table 1) did not show significantly reduced proliferation upon epinephrine stimulation. Another cell line in this group (JVE207) exhibited even increased proliferation. However, as we did not use HT-29 in our study, we cannot make a direct comparison. We also found that epinephrine’s promoting effect on migration tended to be specific for the MSS group in comparison with MSI cells and for *BRAF*-mutated cell lines. As both MSS cell lines and *BRAF*-mutated cells are commonly characterized by a more migratory phenotype compared with MSI and *BRAF* WT cells, respectively,^36^^,^^37^^,^^38^^,^^39^ our data suggest that epinephrine is able to enhance the migratory capacity of CRC cells already prone to migrate or metastasize. Interestingly, it has also been shown that epinephrine can give rise to chemotherapy resistance in CRC,^35^^,^^40^ which we did not explore in this study.

Other neural signals caused a similar effect on the migration potential of molecular subtypes of CRC cells. The same migration induction was observed for GDNF, and here it was further extended to the *KRAS*-mutated cell lines, which are also known to be associated with enhanced migration compared with unmutated CRC cell lines.^41^^,^^42^^,^^43^ GDNF is a neurotrophin conditionally secreted by enteric glia, which are present in the tumor stroma. GDNF might contribute to mucosal homeostasis by supporting epithelial barrier function.^8^^,^^44^ At the same time, GDNF has been shown to increase the migration and proliferation of epithelial cells, which is beneficial in the context of healing but could be unfavorable for CRC patients.^44^^,^^45^^,^^46^ In our study, GDNF induced the colony-formation potential of CRC cells, indeed pointing to a tumor-promoting function in CRC. Notably, GDNF induced pronounced phenotypic effects despite a very low baseline expression of its canonical receptors in most CRC cell lines. Only GFRA1 and GFRA3 expression could be extracted from the transcriptomic analyses, with the levels of GFRA2, a common binding partner for GDNF, and GFRA4 remaining unknown.^47^ This suggests that neural responsiveness may not depend solely on receptor abundance but could involve low-level expression in responsive subpopulations and engagement of non-canonical signaling pathways. These observations underscore the complexity of neural-tumor communication and indicate that the functional outcomes cannot be inferred from receptor expression alone.

Another signaling molecule that is released by enteric glial cells, PGE2, conferred the same response as GDNF by upregulating clonogenicity and migration in CRC cells. PGE2 was previously shown to enhance colorectal tumorigenesis,^48^^,^^49^^,^^50^ and it has been specifically linked to enteric glia as the secretion of PGE2 from tumor-associated enteric glia induced cancer stem cell expansion and tumor growth.^7^ When differentiating patient subtypes based on their genomic profile, subtle increases in migration were observed in MSS and *KRAS*-mutant cell lines compared with MSI and *KRAS* WT CRC, respectively, as well as a tendency to increase cell viability and decrease colony-formation capacity for *BRAF*-mutated cells compared with the unmutated cohort. Although not statistically significant, these results suggest that the response to PGE2 may be specific to both the tumor and patient, potentially influenced by the genomic characteristics of the tumor from which the cells were derived.

Acetylcholine, an excitatory neurotransmitter that acts on nicotinic and muscarinic receptors to regulate essential gut functions, is released from both extrinsic parasympathetic and intrinsic enteric neurons. In our experiments, cholinergic agonists caused an enhancement in clonogenicity and migration of CRC cells. Interestingly, the effect was influenced by both the genomic subtype of the cells as well as the cholinergic receptor subtype, as nicotinic and muscarinic agonists conferred different effects. In CRC literature, muscarinic signaling has received most attention. Muscarinic receptor M3R signaling promotes proliferation, migration, and invasion in CRC.^51^^,^^52^ Knowledge on the involvement of nicotinic signaling in CRC is limited, although it has been shown to induce tumor growth as well.^53^ Both nicotinic and muscarinic stimulation induced a minor decrease in cell viability in *BRAF* WT cells compared to *BRAF*-mutated lines. However, only the muscarinic agonist bethanechol enhanced clonogenicity of *BRAF* WT cells, while the nicotinic agonist DMPP tended to increase migration specifically in *KRAS*-mutated cell lines, suggesting that nicotinic and muscarinic cholinergic signaling differentially affect CRC genomic subtypes. As cholinergic signaling is partly implicated in the same pathways, e.g., Akt, ERK, and EGF, that are affected by *BRAF*,^28^ this might explain differences in the response of *BRAF* WT and -mutated cells to cholinergic signals and will be important for treatment considerations.

We recently identified that among the neuronal processes present in the tumor stroma, the vast majority is VIP-positive, suggesting that VIPergic neurons can directly communicate with tumor cells in CRC patients.^6^ Interestingly, besides the overall colony-formation and migration promoting effects of VIP, it was the only neural signal that significantly increased cell viability of *BRAF*-mutated cell lines compared with *BRAF* unmutated lines, while it did not induce any other mutation-specific responses. Other studies have provided evidence for a tumor-promoting role of VIP both *in vitro* by stimulating cell proliferation^54^ and *in vivo* by enhancing azoxymethane-induced colorectal tumors in rats.^55^ Strikingly, VIP signaling in CRC was shown to affect the *BRAF*-ERK pathway and might, therefore, differentially influence *BRAF*-mutated versus *BRAF* WT cells,^54^ which is in line with our results for cell viability.

Because all neural cues consistently enhanced migration in MSS cells and modestly in *BRAF*-mutated cells, we reasoned that the intrinsic differences between CRC subtypes likely determine their responsiveness to neural signaling. Transcriptomic profiling of untreated cells, therefore, served as a strategy to identify cellular programs that may prime tumor cells for neural cue-induced phenotypes. In MSS cells, enrichment of pathways linked to ECM interaction, lipid metabolism, and PPAR signaling points to a cellular state characterized by metabolic flexibility and structural plasticity—features that are well suited to support migration without invoking a classical EMT program.^56^^,^^57^^,^^58^ In contrast, *BRAF* mutation status was found to be associated with more subtle shifts in metabolic and membrane-related pathways, consistent with more modest and condition-specific phenotypes observed in our functional assays. Notably, neural receptor expression did not align with phenotypic outcomes, supporting a model in which neural cues act by modulating permissive cellular states, rather than through dominant receptor-driven mechanisms. Although this approach could not resolve dynamic transcriptional responses to neural receptor stimulation, these findings suggest that neural regulation of CRC behavior is shaped by core metabolic-ECM programs, highlighting the importance of tumor-intrinsic context when evaluating neural influences on cancer progression.

In this study, we used an *in vitro* approach to enable direct assessment of the effects of defined neural cues on CRC cells. Most studies investigating neural cues in CRC have focused on general tumor-promoting or -suppressive effects. However, CRC is a highly heterogeneous disease, with distinct patient subtypes that differ markedly in prognosis, treatment response, and clinical outcome. Understanding how these different CRC subtypes respond to defined neural signals is, therefore, essential and should inform future mechanistic *in vivo* research. Because multiple factors in the colonic TME, such as the immune system, fibroblasts, and intestinal microbiota, critically contribute to CRC responses,^59^^,^^60^^,^^61^^,^^62^ future studies are necessary to understand how our findings translate in the heterogeneous colonic TME context.

In conclusion, we studied the direct effect of neural signals on different CRC patient subtypes, using an *in vitro* approach. Our results demonstrated that factors commonly secreted by neurons and enteric glia can influence CRC cell clonogenicity, viability, and migration. While most neural cues enhanced colony formation and migration, epinephrine had a partial opposite effect, which reduced both clonogenicity and viability. These distinct responses underscore the importance of a better understanding of neural influences on CRC and their potential as therapeutic targets. Despite high inter-cell line variability, our findings suggest that genomic subtypes, such as MSI and *KRAS* and *BRAF* mutations, may modulate responses to neural signals. Larger patient cohorts and additional influencing factors should be considered in future studies. Collectively, our data highlight the relevance of patient stratification and the need for careful selection of neural targets in CRC therapy development.

### Limitations of the study

This study has several limitations. First, our findings are based on *in vitro* assays using patient-derived CRC cell lines, which enable a controlled assessment of direct neural cue effects but do not capture the full complexity of the TME. Furthermore, while neural signaling *in vivo* is dynamic and context dependent, our experimental design relied on exposure to defined concentrations of individual neural molecules, which may not fully reflect physiological gradients, temporal dynamics, or combinatorial signaling effects. Also, although we included a panel of CRC cell lines representing distinct genomic subtypes, the number of models within each subgroup remained limited and may not encompass the full heterogeneity observed in patients. This may also contribute to variability in responses and limit the generalizability of subtype-specific conclusions. Finally, while we observed functional effects of neural cues on clonogenicity, viability, and migration combined with underlying transcriptional differences in responding and non-responding cell lines, the underlying molecular mechanisms and signaling pathways were not directly interrogated, and causal links between receptor expression and phenotypic outcomes remain unresolved.

## Resource availability

### Lead contact

Requests for further information and resources should be directed to and will be fulfilled by the lead contact, Veerle Melotte (veerle.melotte@maastrichtuniversity.nl).

### Materials availability

This study did not generate new unique reagents.

### Data and code availability

• All data reported in this paper will be shared by the lead contact upon reasonable request.
• This paper does not report original code
• Any additional information required to reanalyze the data reported in this paper is available from the lead contact upon request.

## Acknowledgments

This study was supported by a BOF-mandate at 10.13039/501100009550Hasselt University (BSFWBIOMED) and Maastricht University Medical Centre+ (M.S.T.), Kankeronderzoeksfonds Limburg (KOFL) (M.S.T.), Het Fonds voor Wetenschappelijk Onderzoek – Vlaanderen (FWO) long-term travel grant (V472323N to M.S.T.), and the 10.13039/501100003246Netherlands Organisation for Scientific Research VIDI grant (09150172110100 to V.M.).

## Author contributions

V.M., W.B., and M.S.T. designed the study; K.M.S. advised in cohort selection; A.B. and V.M. supervised the study; A.B. provided the patient-derived cell lines for this study; M.S.T. performed the experiments and collected the data; R.C. assisted in experimental setup and techniques; G.C. performed bioinformatic analyses, and M.S.T. performed the other analyses; K.M.S. assisted in the statistical analyses; M.S.T., W.B., and V.M. drafted the manuscript. All authors have assisted with result interpretation and revised the manuscript.

## Declaration of interests

A.B. received research support from Neophore, AstraZeneca, and Boehringer Ingelheim outside the current study; he is a shareholder of NeoPhore and Kither Biotech and a member of the Scientific Advisory Board of NeoPhore.

## STAR★Methods

### Key resources table

**Table:** 

REAGENT or RESOURCE	SOURCE	IDENTIFIER
**Chemicals, peptides, and recombinant proteins**		

Dimethylphenylpiperazinium (DMPP)	Sigma	Cat#D5891
Bethanechol	Sigma	Cat#C5259
Epinephrine	Sigma	Cat#324900
Vasoactive intestinal peptide (VIP)	Sigma	Cat#05-23-2101
Glial cell line-derived neurotrophic factor (GDNF)	Peprotech	Cat#450-10
Prostaglandin E2 (PGE2)	Cayman Chemical	Cat#14010

**Critical commercial assays**		

CellTiter-Glo Luminescent Cell Viability assay	Promega	Cat#G7570
GenePrint® 10 System (10-Locus STR System for Cell Line Authentication)	Promega	Cat#B9510

**Deposited data**		

dbSNP v153	NCBI	https://www.ncbi.nlm.nih.gov/snp/
GENCODE v44	GENCODE	https://www.gencodegenes.org/
hg38 (GRCh38 human genome build)	Genome Reference Consortium	https://www.ncbi.nlm.nih.gov/grc/human
KEGG	Kyoto University	https://www.genome.jp/kegg/

**Experimental models: Cell lines**		

CACO2	IFOM biobank	CVCL_0025
COCM1	IFOM biobank	CVCL_1127
COGA12	IFOM biobank	CVCL_A073
COLO320	IFOM biobank	CVCL_1989
CRC0781_XL	IFOM biobank	Lazzari et al.^63^
CRC1360_XL	IFOM biobank	Livio Trusolino
DIFI	IFOM biobank	CVCL_6895
HCA46	IFOM biobank	CVCL_2468
HCA7	IFOM biobank	CVCL_0289
HDC143	IFOM biobank	CVCL_A384
HDC54	IFOM biobank	CVCL_A362
HROC126	IFOM biobank	CVCL_1D12
HROC334	IFOM biobank	CVCL_1D18
HROC57	IFOM biobank	CVCL_1G03
JVE103	IFOM biobank	CVCL_EG19
JVE114	IFOM biobank	CVCL_EG21
JVE207	IFOM biobank	CVCL_EG25
JVE528	IFOM biobank	CVCL_EG31
KM12C	IFOM biobank	CVCL_9547
KP7038T	IFOM biobank	CVCL_EG35
RW7213	IFOM biobank	CVCL_D175
SNU977	IFOM biobank	CVCL_5109
SW1417	IFOM biobank	CVCL_1717
SW1463	IFOM biobank	CVCL_1718

**Software and algorithms**		

STAR	Dobin et al.^64^	https://github.com/alexdobin/STAR
RSEM	Li and Dewey	https://github.com/deweylab/RSEM
DESeq2	Bioconductor	
iDEP	Ge et al.^65^	https://bioinformatics.sdstate.edu/idep/
ImageJ (v1.52p)	NIH	https://imagej.nih.gov/ij/
Incucyte Scratch Wound Analysis Software Module	Sartorius	Included with Incucyte system
GraphPad Prism 8	GraphPad software	Version 8.0.2, https://www.graphpad.com/

**Other**		

NovaSeq 6000 Squencing System	Illumina	https://www.illumina.com/systems/sequencing-platforms/novaseq.html
EnVision 2104 Multilabel Reader	PerkinElmer	Model 2104
Incucyte CX3 Live-Cell Analysis System	Sartorius	https://www.sartorius.com/en/products/live-cell-imaging-analysis/live-cell-analysis-instruments
Incucyte WoundMaker 96-tool	Sartorius	Cat#4563

### Experimental model and study participant details

#### Patient-derived CRC cell lines

Patient-derived CRC cell lines were obtained from the IFOM biobank and were all tested for mycoplasma and STR cell line authenticated using the GenePrint 10 System.^66^ Information about patient and tumor characteristics, including age, sex, tumor location, microsatellite status, mutational profile (e.g., *KRAS* and *BRAF* mutations), were available. These data together with culture conditions were used to select specific cohorts of cell lines to study the difference between MSS and MSI, *KRAS* wildtype (WT) and mutated (MUT), and *BRAF* WT and mutated cell lines. Cells were cultured in DMEM high glucose or RPMI 1640 media (EuroClone) supplemented with 10% fetal calf serum (FCS), 1% L-glutamine and 1% Pen/Strep and maintained at 37°C and 5% CO_2_. Cell lines were first expanded and kept until stable culture conditions could be ensured before experimental use. If these could not be reached, cell lines were excluded from the experiments, which let to drop-outs from the originally selected cohorts of five cell lines. Only cell lines that were successfully used in the experiments are described in the results section.

### Method details

#### Genetic and transcriptomic profiling of cell lines

Next-generation sequencing (NGS) libraries for DNA were prepared according to established workflows and manufacturer’s protocols.^67^^,^^68^ Libraries were pooled and sequenced on the NovaSeq 6000 platform (Illumina Inc., San Diego, CA, USA). Raw sequencing reads (FASTQ files) were processed as previously reported.^69^ Cell line identity was confirmed using SNP_ID profiles (dbSNP v153), retaining alleles with >30% abundance and requiring >95% concordance.^69^^,^^70^ Mutation calling was performed as previously described.^67^^,^^69^ Total RNA was extracted using standard procedures and manufacturer protocols.^68^^,^^71^^,^^72^ RNA-seq reads were aligned to the human reference genome (hg38) using STAR. Transcript and gene-level quantification was performed with RSEM, using GENCODE v44 as the gene annotation reference. Gene-level expression values were normalized and converted to robust FPKM values using DESeq2.^68^^,^^71^^,^^72^ Differential gene expression and pathway analyses were performed using the iDEP platform,^65^ which applies established statistical methods for transcriptomic analysis. Gene expression values were filtered and normalized within iDEP, followed by differential expression analysis using a DESeq2-based framework. Genes with an absolute fold change ≥2 and a false discovery rate (FDR) < 0.25 were considered differentially expressed. Gene set enrichment analysis (GSEA) was performed using KEGG pathway annotations to identify coordinated pathway-level differences between experimental groups based on enrichment scores and adjusted *p*-values.

#### Neural signaling molecule treatments

Concentration ranges for the neural molecules dimethylphenylpiperazinium (DMPP), bethanechol, epinephrine, vasoactive intestinal peptide (VIP), glial cell line-derived neurotrophic factor (GDNF) and prostaglandin E2 (PGE2) were obtained from literature and tested in a pilot experiment with an MSS and MSI cell line. Both cell lines were plated in 96-well plates and treated in triplicate with the concentrations depicted in Figure S2 in a total volume of 100 μL. After two and five days, CellTiter-Glo luminescent assay (Promega) was performed as by manufacturer’s instructions using EnVision 2104 multilabel reader (PerkinElmer). The following concentrations were selected for treatment in further experiments: DMPP 50 μM, bethanechol 100 μM, epinephrine 200 μM (high) and 20 μM (low), VIP 100 nM, GDNF 50 ng/mL, PGE2 5 μM.

#### Cell viability assay

18,000 cells/well were plated in 96-well plates in a volume of 50 μL. Cells were subsequently treated with 50 μL of the neural cues diluted in culture medium to obtain a final volume of 100 μL with the final concentrations of the neural molecules as specified above. All conditions were plated in triplicate. After two and five days of incubation, plates were subjected to a CellTiter-Glo luminescent assay (Promega) to study short-term metabolic viability as by manufacturer’s instructions and readout using EnVision 2104 multilabel reader (PerkinElmer).

#### Colony formation assay

To obtain single cells for the colony formation assay, which reflects tumor-initiating potential, cells were resuspended and plated in a low density of 250 cells/well in 48-well plates in a volume of 100 μL. Treatments were applied similarly as for the cell viability assay with a volume of 100 μL to obtain a total volume of 200 μL. All conditions were plated in triplicate. After two weeks of incubation, cells were fixed with methanol for 20 min and subsequently stained with 0.4% Crystal violet in MilliQ water for 30 min while shaking. Plates were imaged using a high-resolution scanner and images were analyzed using colony area and colony measurer plugins in ImageJ 1.52p software (NIH, Bethesda, MD).

#### Migration assay

To prepare cells for the scratch assay and asses cell migration, cells were plated in Incucyte Imagelock 96 well plates (Sartorius) and incubated until a fully confluent monolayer could be observed. For some cell line, growth characteristics did not enable a confluent monolayer, so these cell lines were excluded for this assay. When wells were confluent, scratching was carried out using an Incucyte Wound Maker 96-Tool (Sartorius), medium with detached cells was removed and cells were treated with the neural cues as described before in a total volume of 100 μL. Plates were placed in an Incucyte machine (Sartorius) and pictures were made every 12 h for a duration of 84–120 h. The Incucyte Scratch Wound Analysis Software Module (Sartorius) was used to analyze the images based on relative wound density compared to timepoint 0.

### Quantification and statistical analysis

Data were analyzed by Wilcoxon matched-pairs signed rank test (non-parametric data) for the comparison between untreated and neural-treated groups as the same cells were represented in both groups. Unpaired t tests were applied for the comparison within neural treatment groups between genomic CRC groups. two-way ANOVA with post-test correction (Dunnett’s multiple comparisons test) was used when comparing multiple genomic groups. The specific statistical test is reported in each figure legend. All statistical analyses were performed using GraphPad Prism8 (version 8.0.2).
