# Supplementary material for: Neural cues differentially modulate colorectal cancer cell behavior depending on patients’ genomic background
Source: iScience. 2026 May 28;29(6):116153. doi: 10.1016/j.isci.2026.116153 (PMC13233565; doi:10.1016/j.isci.2026.116153)
Supplement: Document S1. Figures S1–S5 and Tables S1 and S2 [file mmc1.pdf]

iScience, Volume *29*

Supplemental information

Neural cues differentially modulate colorectal cancer cell behavior depending on patients' genomic background

Meike S. Thijssen, Rosaria Chilà, Giovanni Crisafulli, Kim M. Smits, Alberto Bardelli, Werend Boesmans, and Veerle Melotte

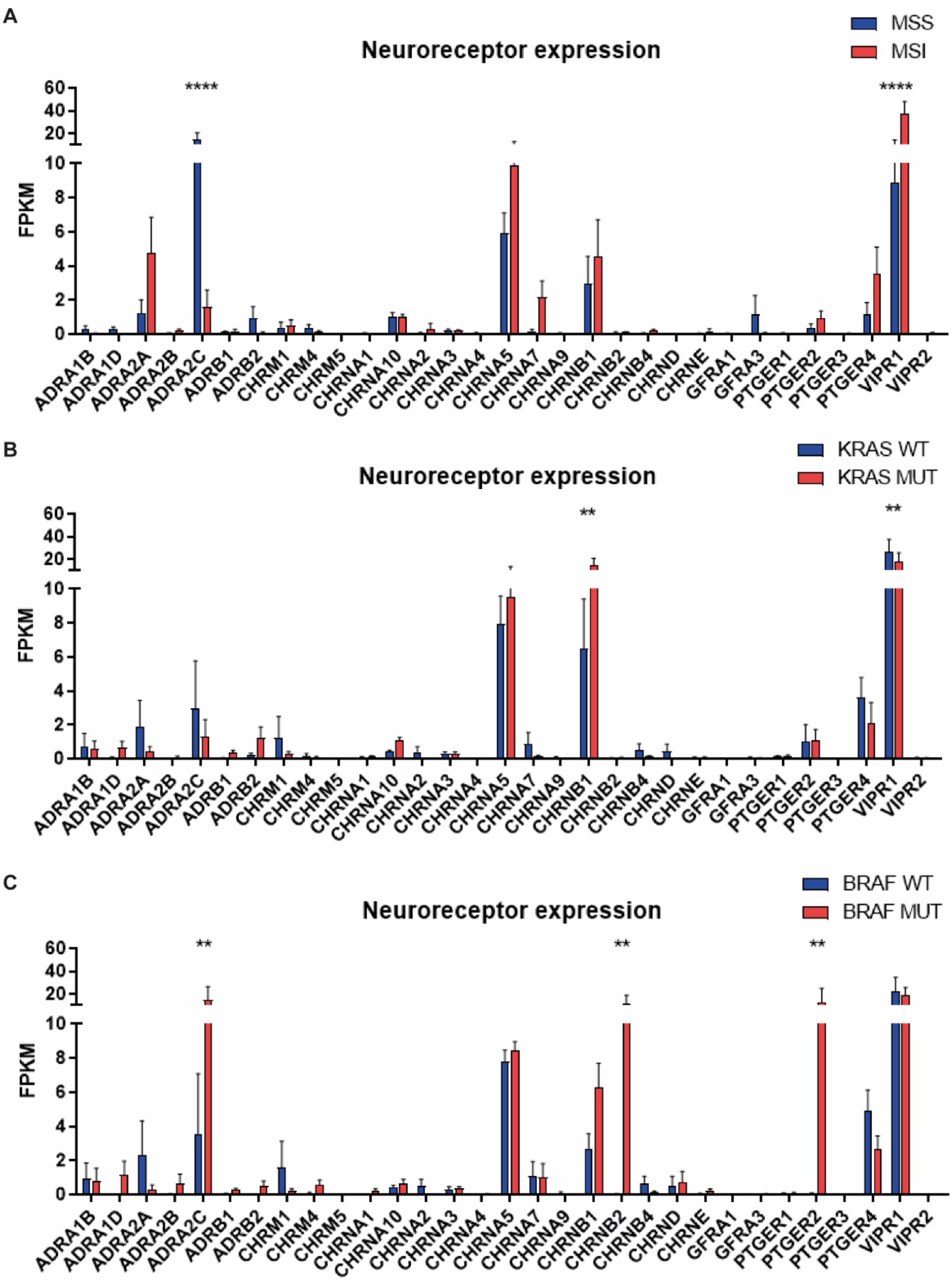

Figure S1. Neuroreceptor expression in the cohorts. (A) Neuroreceptor expression in the cell lines selected for the MSS (n=4) and MSI (n=5) groups shows that these cells express multiple neuroreceptors for the neural cues used in this study and that the expression of ADRA2C and VIPR1 are significantly different between groups. **(B)** *KRAS* mutation status (WT: n=5, MUT: n=5) is significantly linked to alterations in the CHRNB1 and VIPR1 neuroreceptor expression. **(C)** *BRAF* mutated cell lines (n=4) present with an increase in ADRA2C, CHRNB2 and PTGER2 neuroreceptor expression compared to *BRAF* WT cell lines (n=4). The significance level was analyzed by two-way ANOVA to compare the expression of neuroreceptor genes between genomic subgroups of CRC cell lines (* $p<0.05$, ** $p<0.01$).

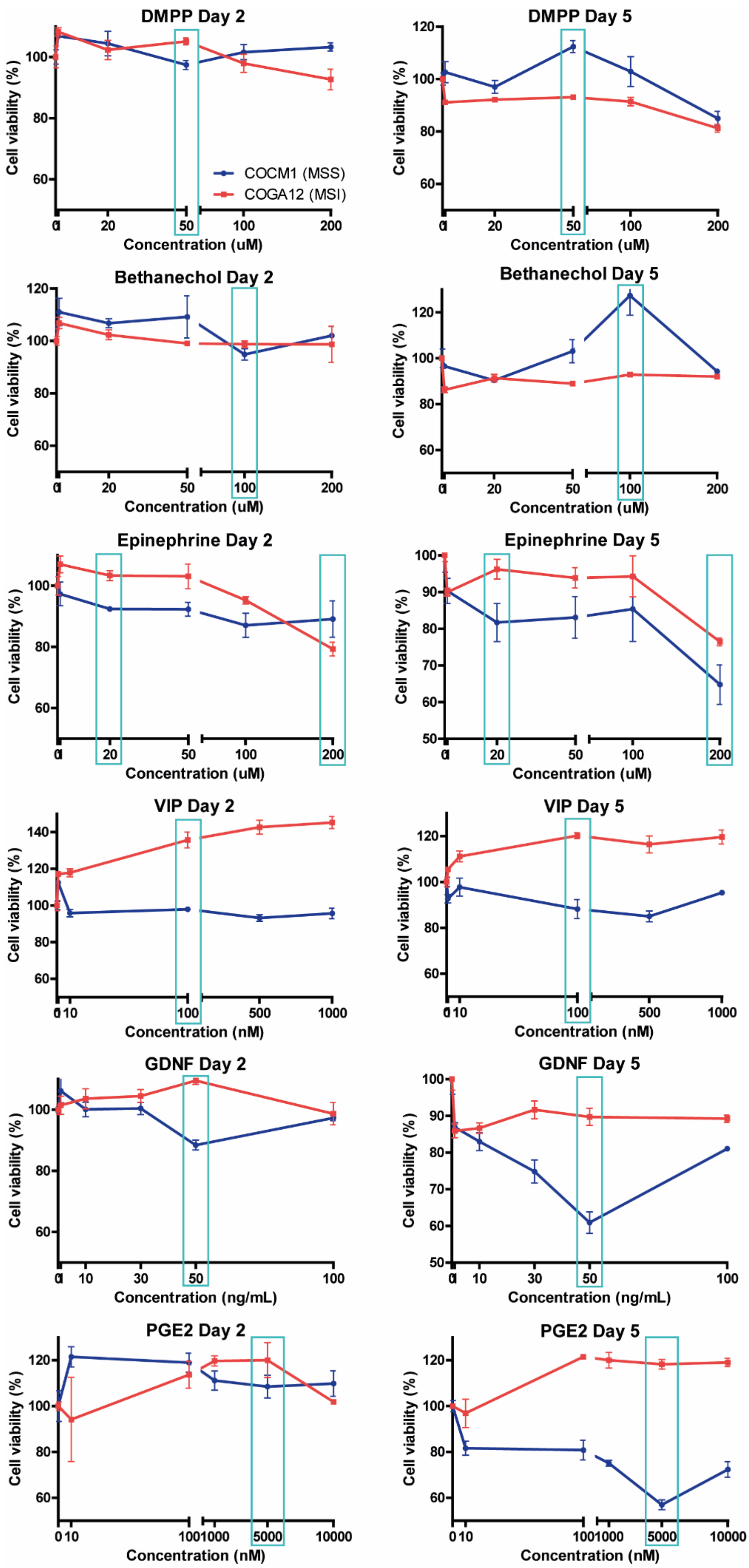

Figure S2. Neural cues concentration determination using two CRC cell lines. An MSS cell line (COCM1) and an MSI cell line (COGA12) were used to test literature-based concentration ranges for the neural signals used in this study. Cells were incubated with the molecules for two and five days and cell viability was measured. The following concentrations were selected for this study: DMPP 50 μM, bethanechol 100 μM, epinephrine 200 μM (high) and 20 μM (low), VIP 100 nM, GDNF 50 ng/mL, PGE2 5 μM.

A

MSS	MSI
Caco2	COGA12
COCM1	HCA7
COLO320	HDC143
DIFI	KM12C
HCA46	KP7038T
HDC54	
HROC126	
JVE103	
JVE114	
SNU977	

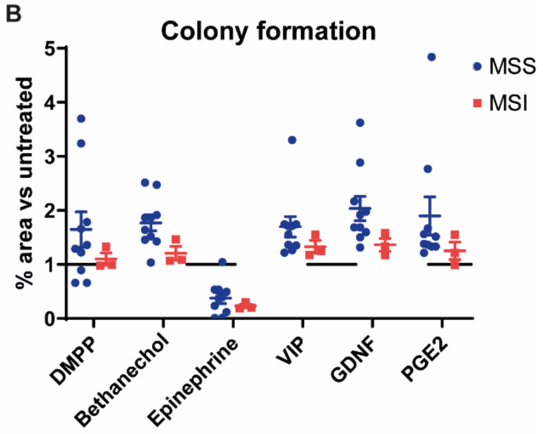

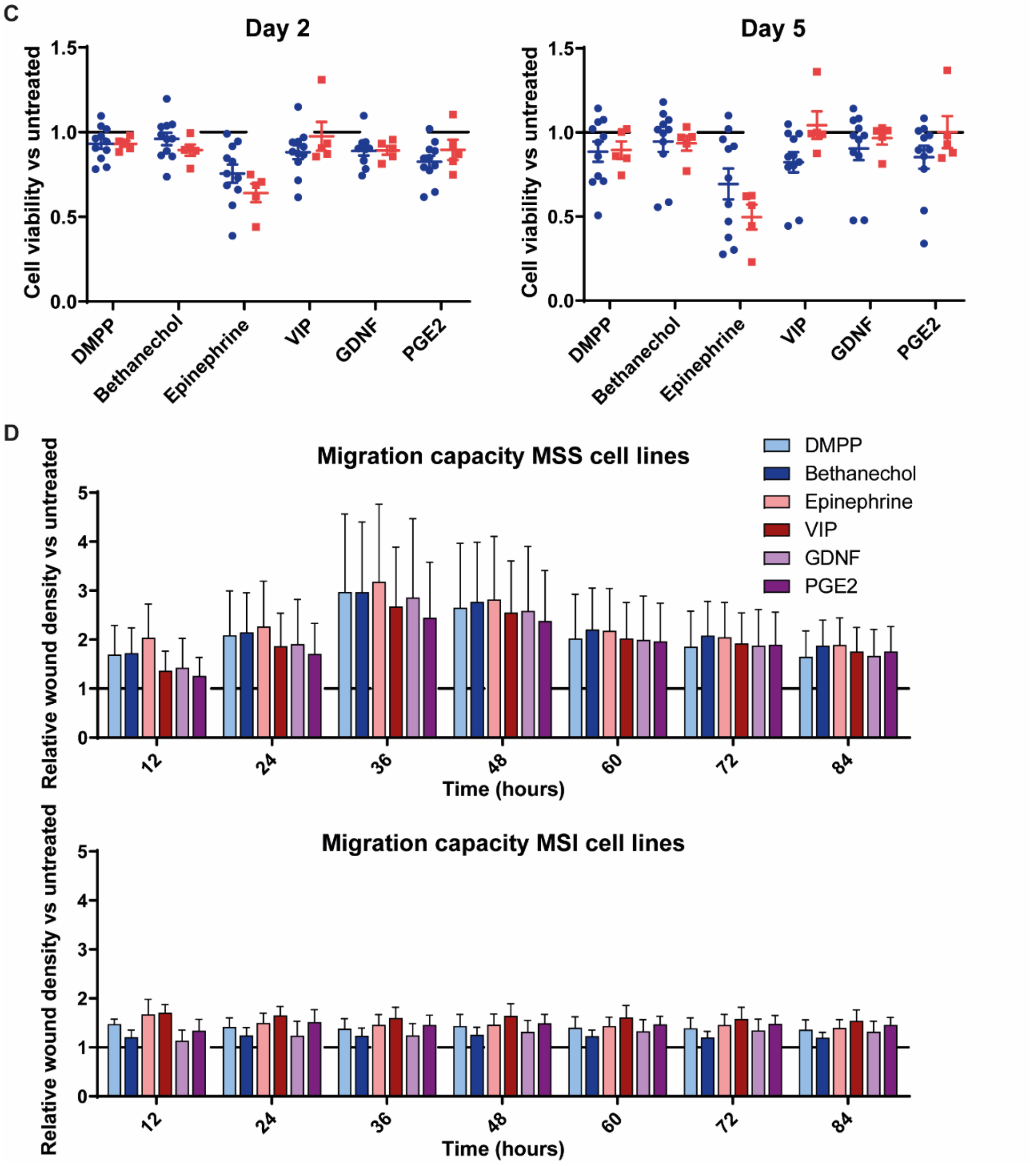

Figure S3| The role of microsatellite status in the response to neural cues. (A) An extended MSS (N=10) and MSI (N=5) cell line cohort are used for this analysis. Similar as in the selected MSS vs MSI cohorts, **(B)** colony formation ability and **(C)** cell viability of CRC cells after neural stimulation are not affected by microsatellite status. **(D)** The trend of increased migration capacity after neural for MSS cell lines is retained although less pronounced in the extended cohort.

A

KRAS WT	KRAS MUT
Caco2	CRC1360_XL
COCM1	HROC334
COLO320	JVE528
DIFI	RW7213
HCA46	SW1463
HDC54	
HROC126	
JVE103	
JVE114	
SNU977	

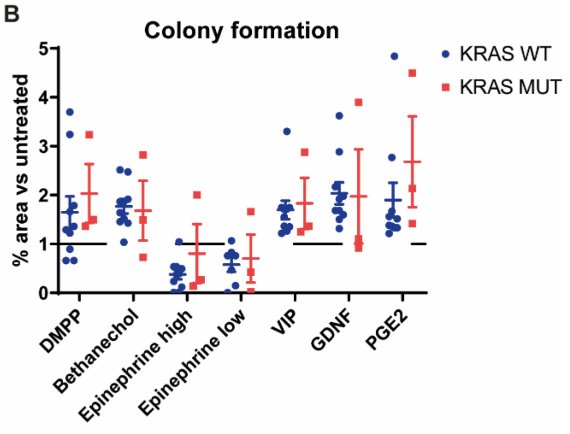

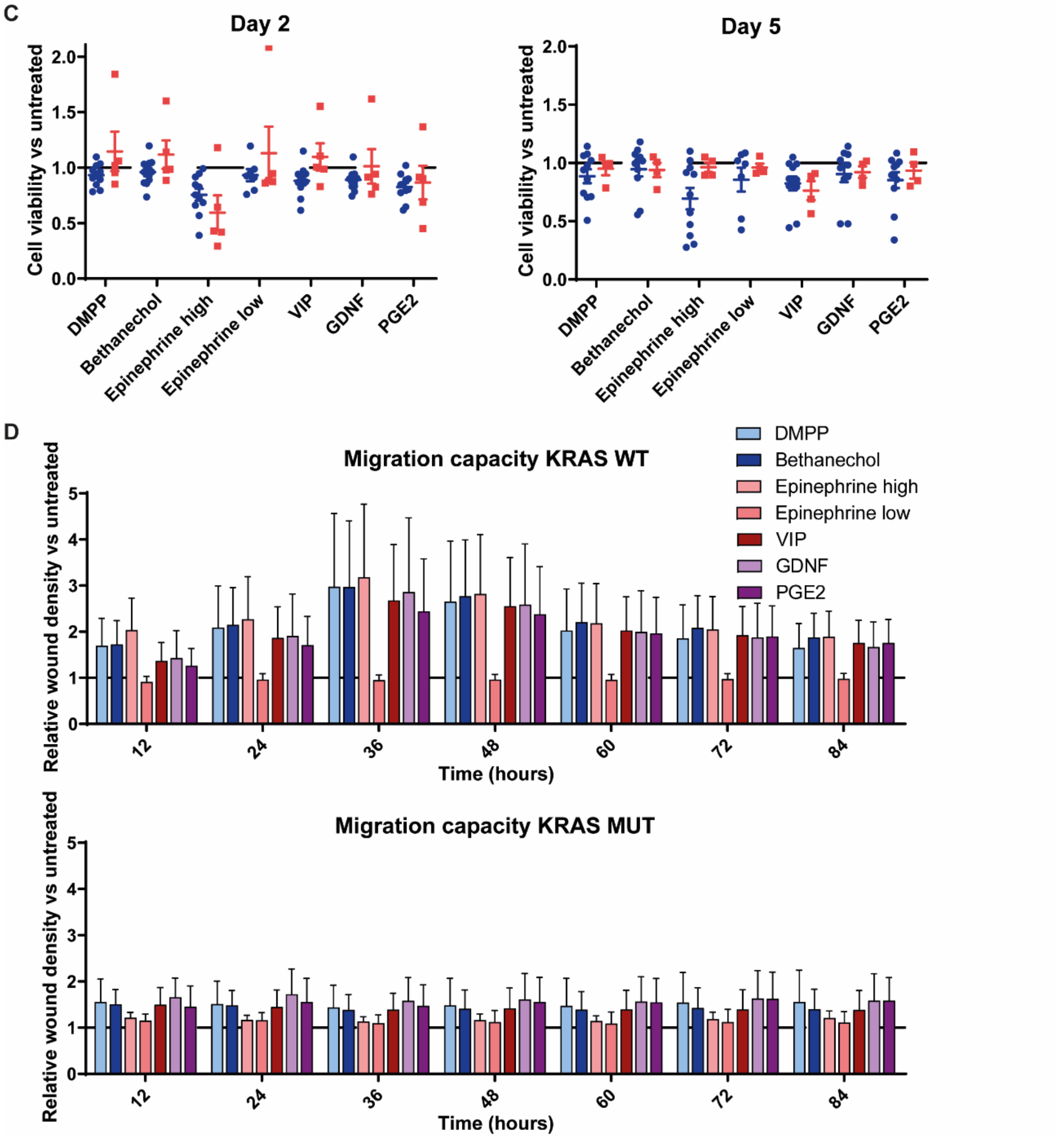

Figure S4. The role of *KRAS* mutation status in the response to neural cues. (A) An extended *KRAS* WT (N=10) and *KRAS* mutated (N=5) cell line cohort are used for this analysis. Similar as for the selected cohorts, *KRAS* mutation status did not alter the neural responses on **(B)** clonogenicity and **(C)** cell viability. **(D)** However, the trend of increased migration in the DMPP, GDNF and PGE2 conditions for *KRAS* mutant cells was lost after extending the cohort.

A

BRAF WT	BRAF MUT
Caco2	CRC0781_XL
COCM1	HROC57
COLO320	JVE207
DIFI	SW1417
HCA46	
HDC54	
HROC126	
JVE103	
JVE114	
SNU977	

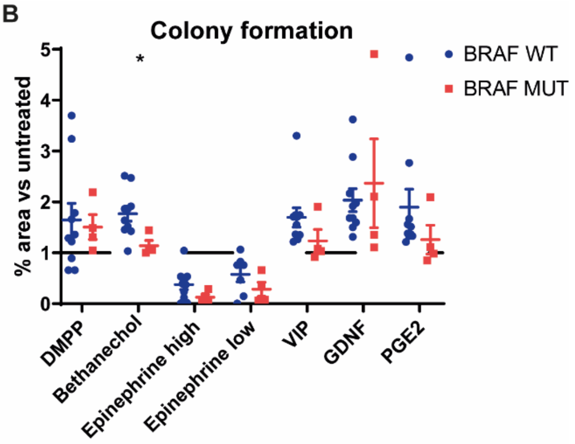

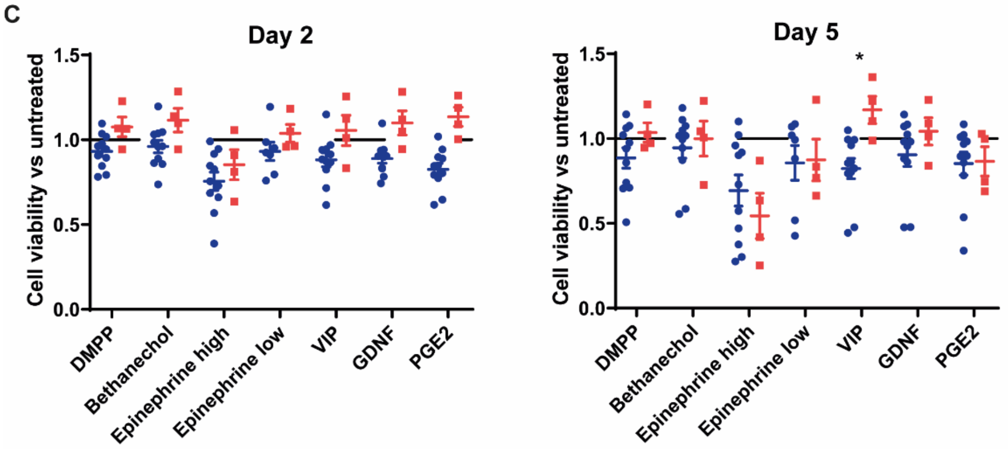

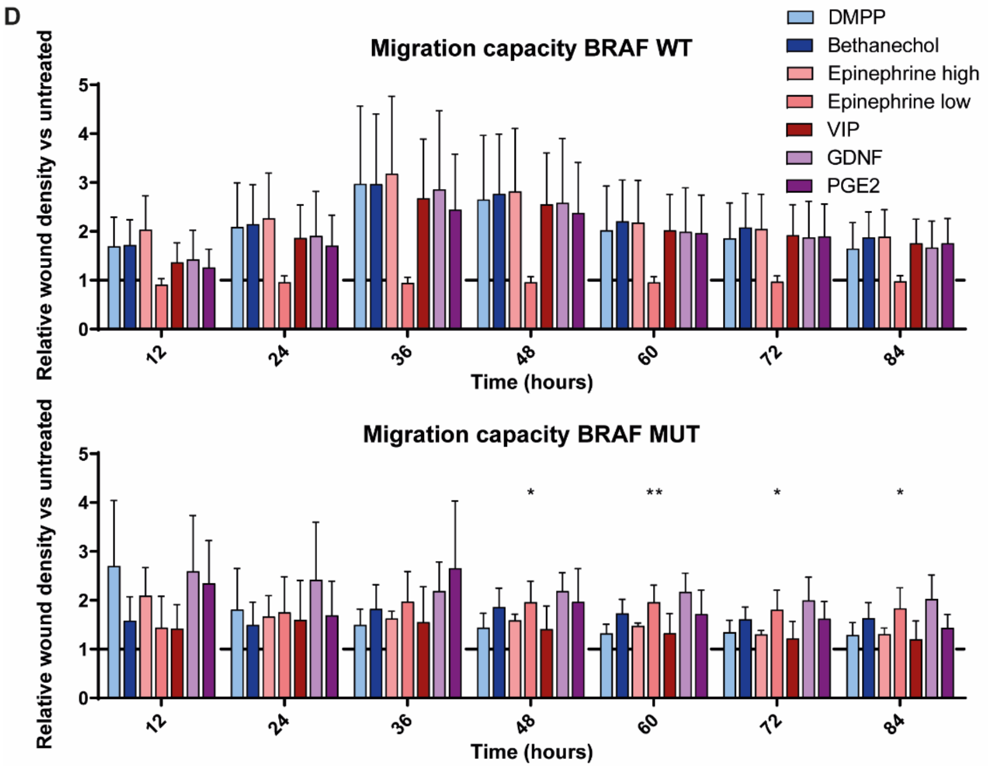

Figure S5. The role of *BRAF* mutation status in the response to neural cues. (A) An extended *BRAF* WT (N=10) and *BRAF* mutated (N=4) cell line cohort are used for this analysis. In this extended cohort, both **(B)** the decreased clonogenicity after bethanechol stimulation, and **(C)** the increased viability after VIP treatment in *BRAF* mutated cell lines were confirmed. **(D)** The increased migration of *BRAF* mutant cell lines was only verified for epinephrine (low) and not for GDNF stimulation.

Table S1. The transcriptomic alterations of microsatellite status.

Direction	GSEA Analysis: MSI-MSS	NES	adj.Pval
Down	ECM-receptor interaction	-0.6078	1.4e-03
	Cholesterol metabolism	-0.5999	6.1e-03
	PPAR signaling pathway	-0.5749	9.2e-03
	Complement and coagulation cascades	-0.5599	9.2e-03
	Basal cell carcinoma	-0.5562	1.1e-02
	Folate biosynthesis	-0.6855	3.1e-02
	Inflammatory bowel disease	-0.5782	6.6e-02
	Viral myocarditis	-0.5233	8.5e-02
	Staphylococcus aureus infection	-0.5429	8.7e-02
Up	Ribosome	0.6211	5.3e-08
	Glycolysis/Gluconeogenesis	0.5791	3.9e-03
	Fatty acid degradation	0.6322	6.1e-03
	Steroid hormone biosynthesis	0.5746	6.1e-03
	Retinol metabolism	0.5227	1.1e-02
	Pyruvate metabolism	0.5773	3.9e-02
	Linoleic acid metabolism	0.625	5.4e-02
	Nitrogen metabolism	0.6864	5.6e-02
	Mucin type O-glycan biosynthesis	0.6378	6.0e-02
	Proximal tubule bicarbonate reclamation	0.6281	8.5e-02
	Tryptophan metabolism	0.5125	8.6e-02

Table S2. The transcriptomic alterations of *BRAF* mutation status.

Direction	GSEA Analysis: BRAFMUT-BRAFWT	NES	adj.Pval
Down	Ascorbate and aldarate metabolism	-0.7749	1.0e-04
	Retinol metabolism	-0.638	4.1e-04
	Pentose and glucuronate interconversions	-0.7086	7.5e-04
	Drug metabolism-cytochrome P450	-0.5763	3.4e-03
	Homologous recombination	-0.5726	8.4e-03
	One carbon pool by folate	-0.5683	5.0e-02
	Nitrogen metabolism	-0.6766	5.8e-02
	Proximal tubule bicarbonate reclamation	-0.6193	7.3e-02
	Fat digestion and absorption	-0.5848	9.0e-02
Up	Staphylococcus aureus infection	0.615	1.0e-02
	Type I diabetes mellitus	0.6562	1.1e-02
	Allograft rejection	0.7093	3.0e-02
	Graft-versus-host disease	0.7209	3.3e-02
	Autoimmune thyroid disease	0.6457	3.6e-02
	Glycosaminoglycan biosynthesis-keratan sulfate	0.7519	4.1e-02
	Inflammatory bowel disease	0.5682	5.4e-02
	Asthma	0.7454	6.3e-02
	Glycosaminoglycan biosynthesis-chondroitin sulfate/dermatan sulfate	0.6133	7.3e-02
	Intestinal immune network for IgA production	0.6128	7.3e-02
	Glycosphingolipid biosynthesis-ganglio series	0.6627	9.1e-02
